# Supplementary material for: Dose- and Segment-Dependent Disturbance of Rat Gut by Ionizing Radiation: Impact of Tight Junction Proteins
Source: Int J Mol Sci. 2023 Jan 16;24(2):1753. doi: 10.3390/ijms24021753 (PMC9863103; doi:10.3390/ijms24021753)

Claudin-1

jejunum

colon

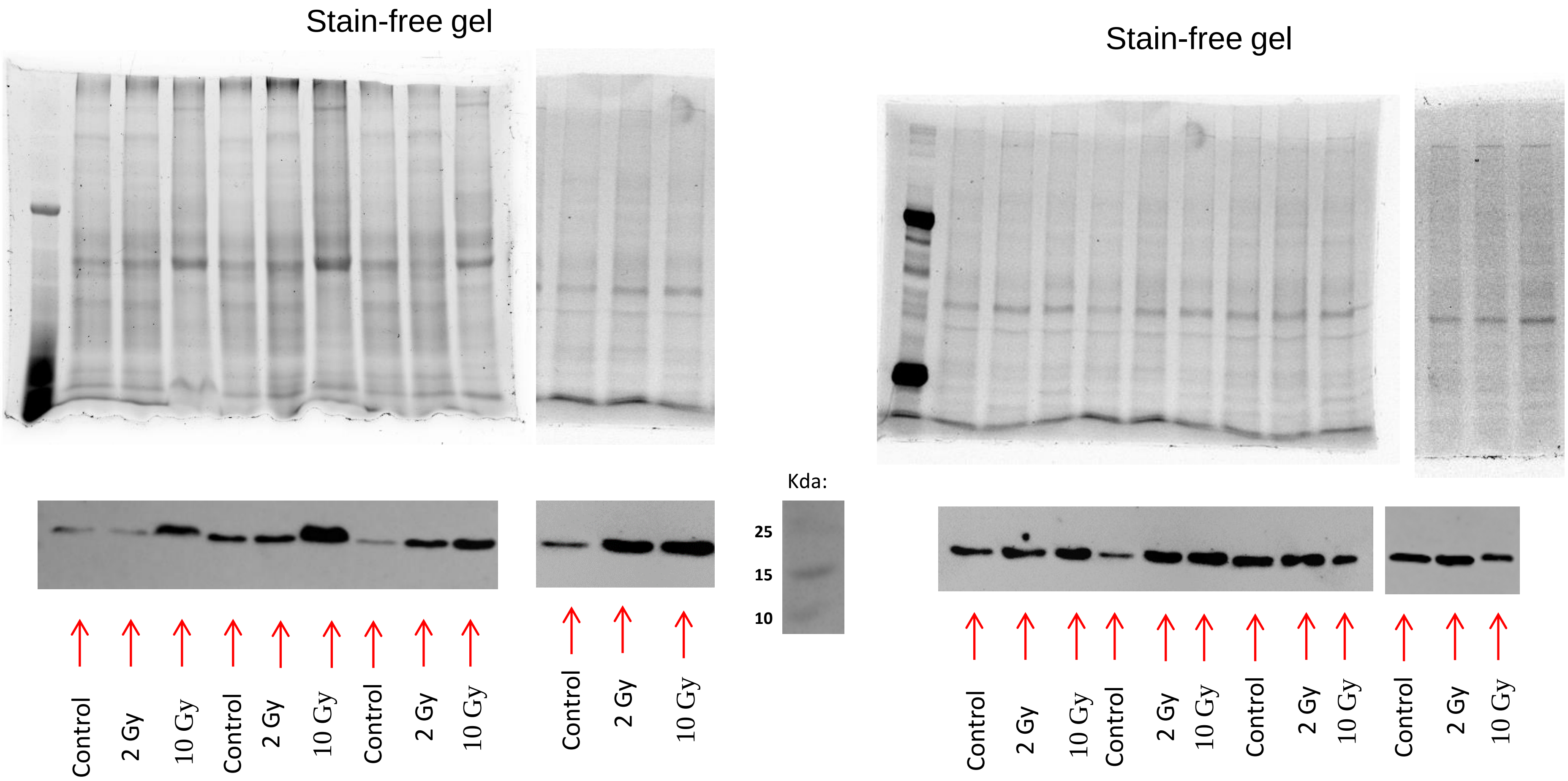

Claudin-2

jejunum

colon

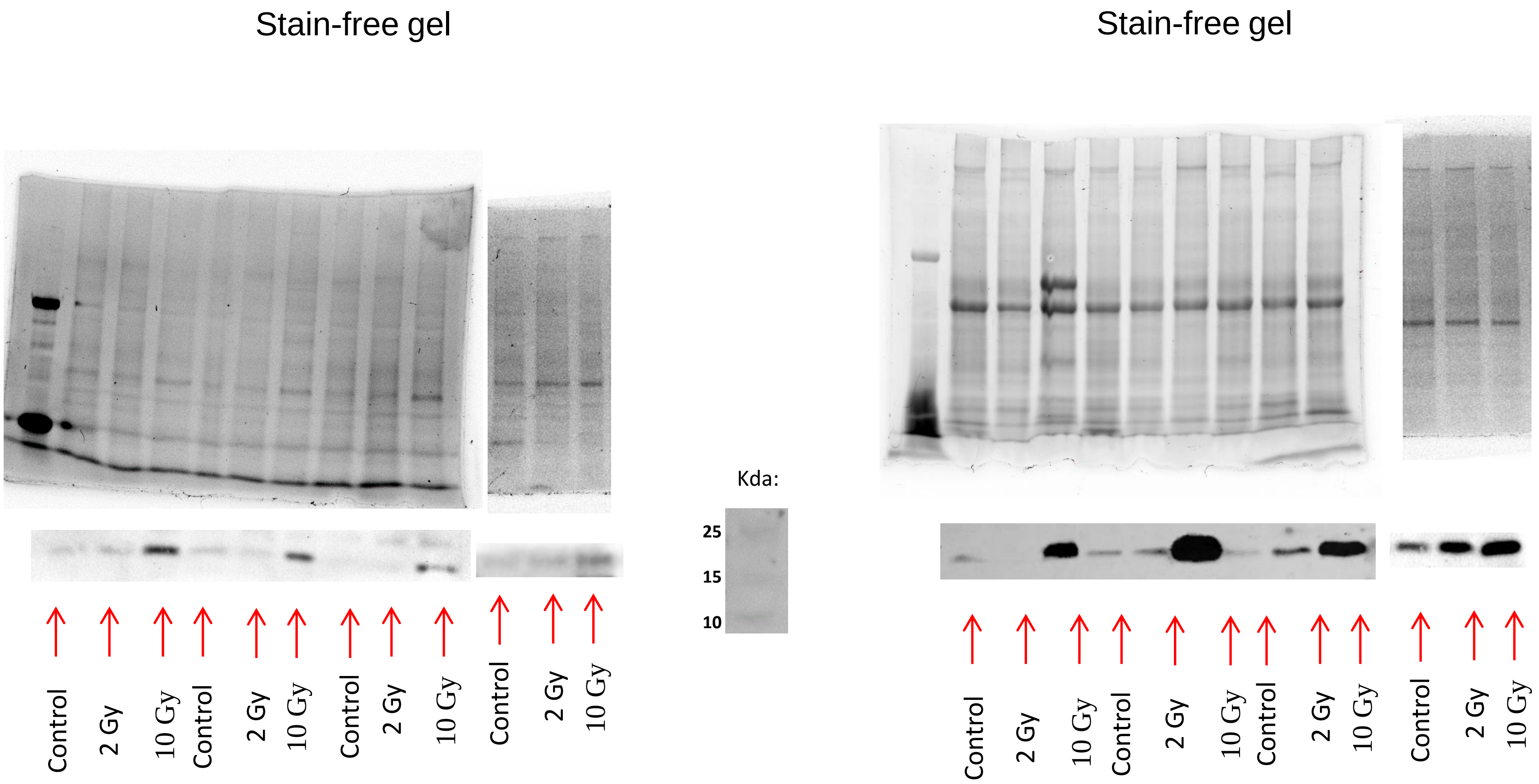

Claudin-3

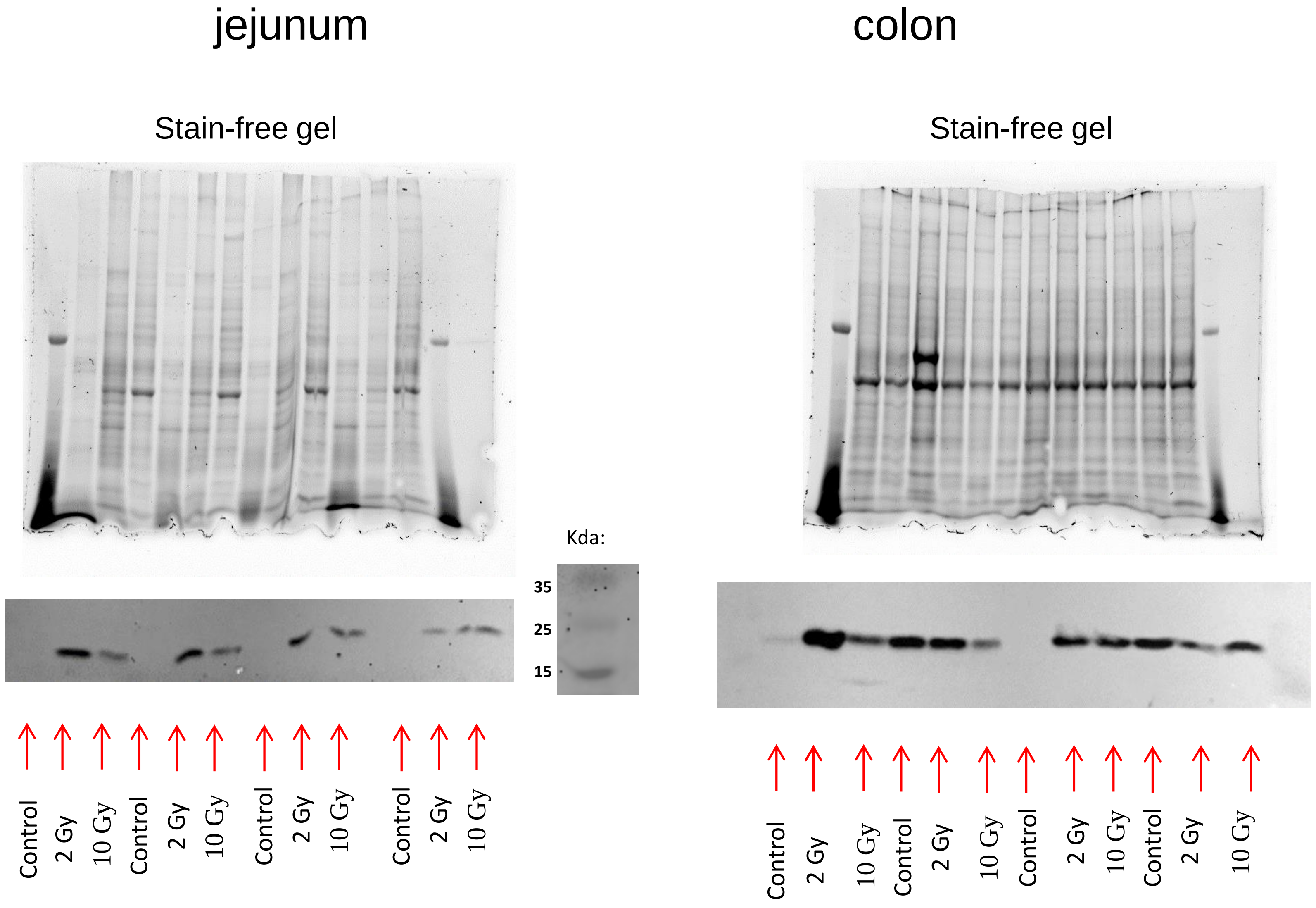
jejunum
colon
Stain-free gel
Stain-free gel
Kda:
35
25
15
Control
2 Gy
10 Gy
Control
2 Gy
10 Gy
Control
2 Gy
10 Gy
Control
2 Gy
10 Gy
Control
2 Gy
10 Gy
Control
2 Gy
10 Gy
Control
2 Gy
10 Gy
Control
2 Gy
10 Gy

Claudin-4

jejunum colon

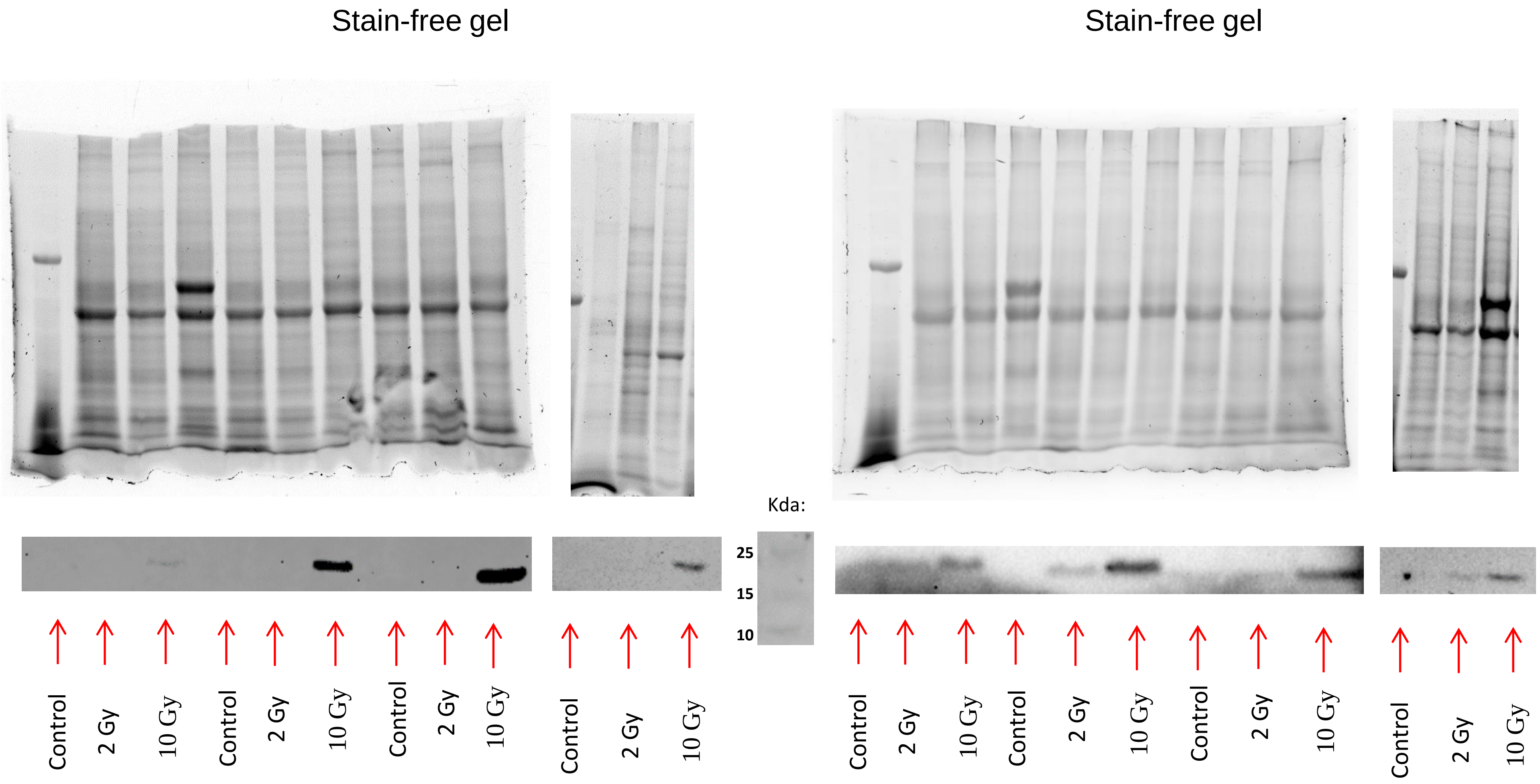

Occludin

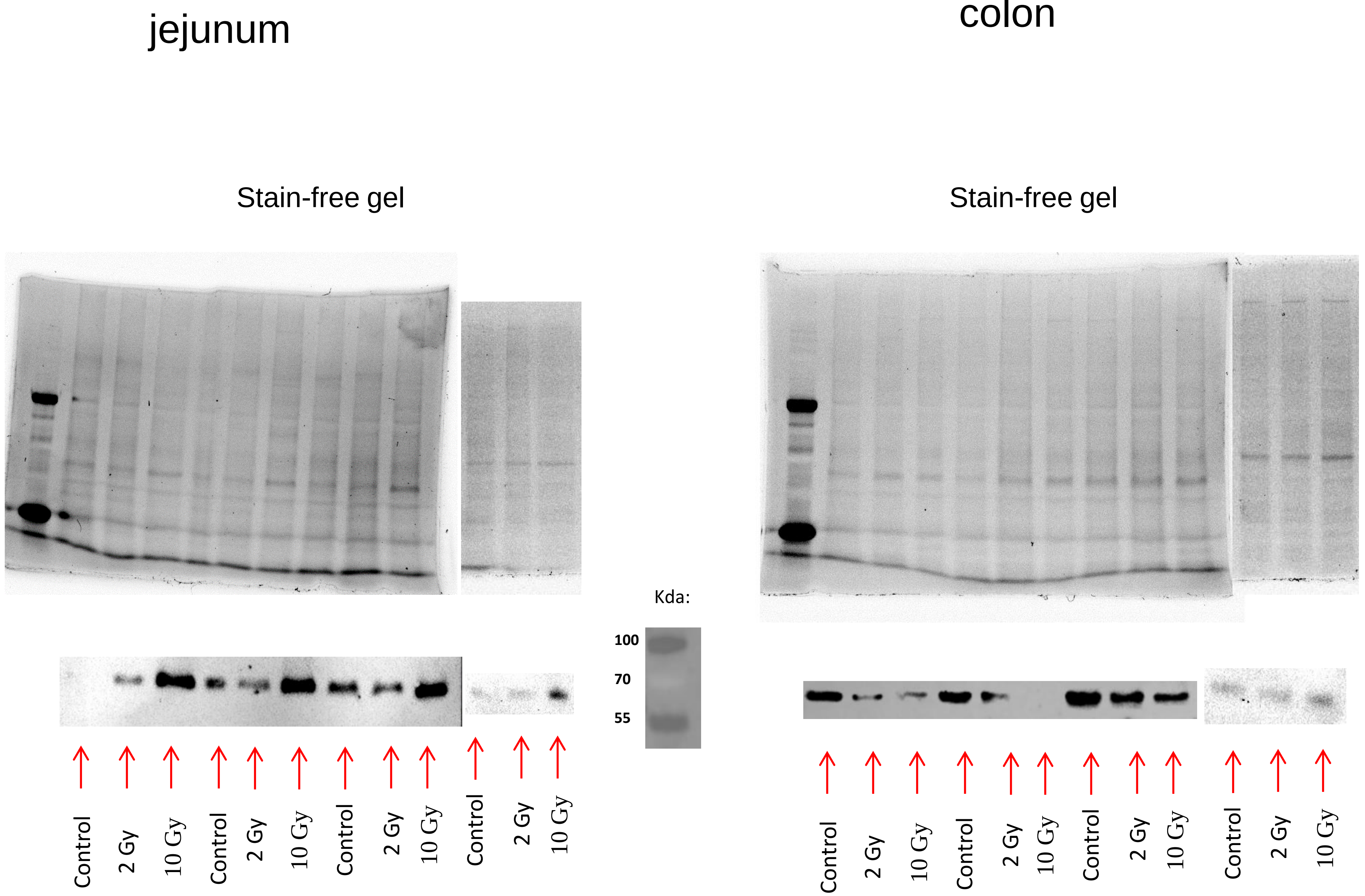
jejunum
colon
Stain-free gel
Stain-free gel
Kda:
100
70
55
Control
2 Gy
10 Gy
Control
2 Gy
10 Gy
Control
2 Gy
10 Gy
Control
2 Gy
10 Gy
Control
2 Gy
10 Gy
Control
2 Gy
10 Gy
Control
2 Gy
10 Gy
Control
2 Gy
10 Gy

Tricellulin

jejunum

colon

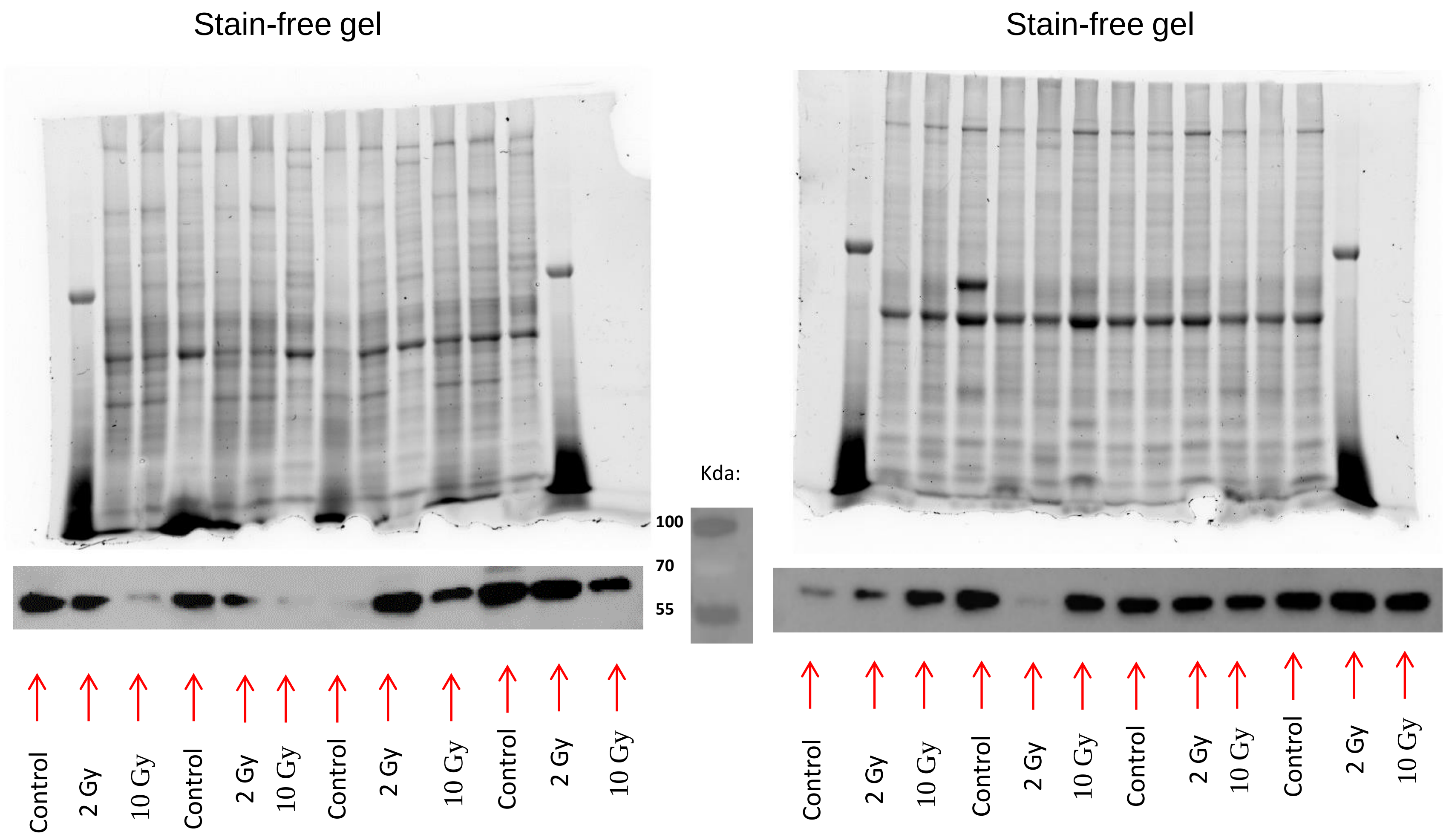

Cleaved caspase-3

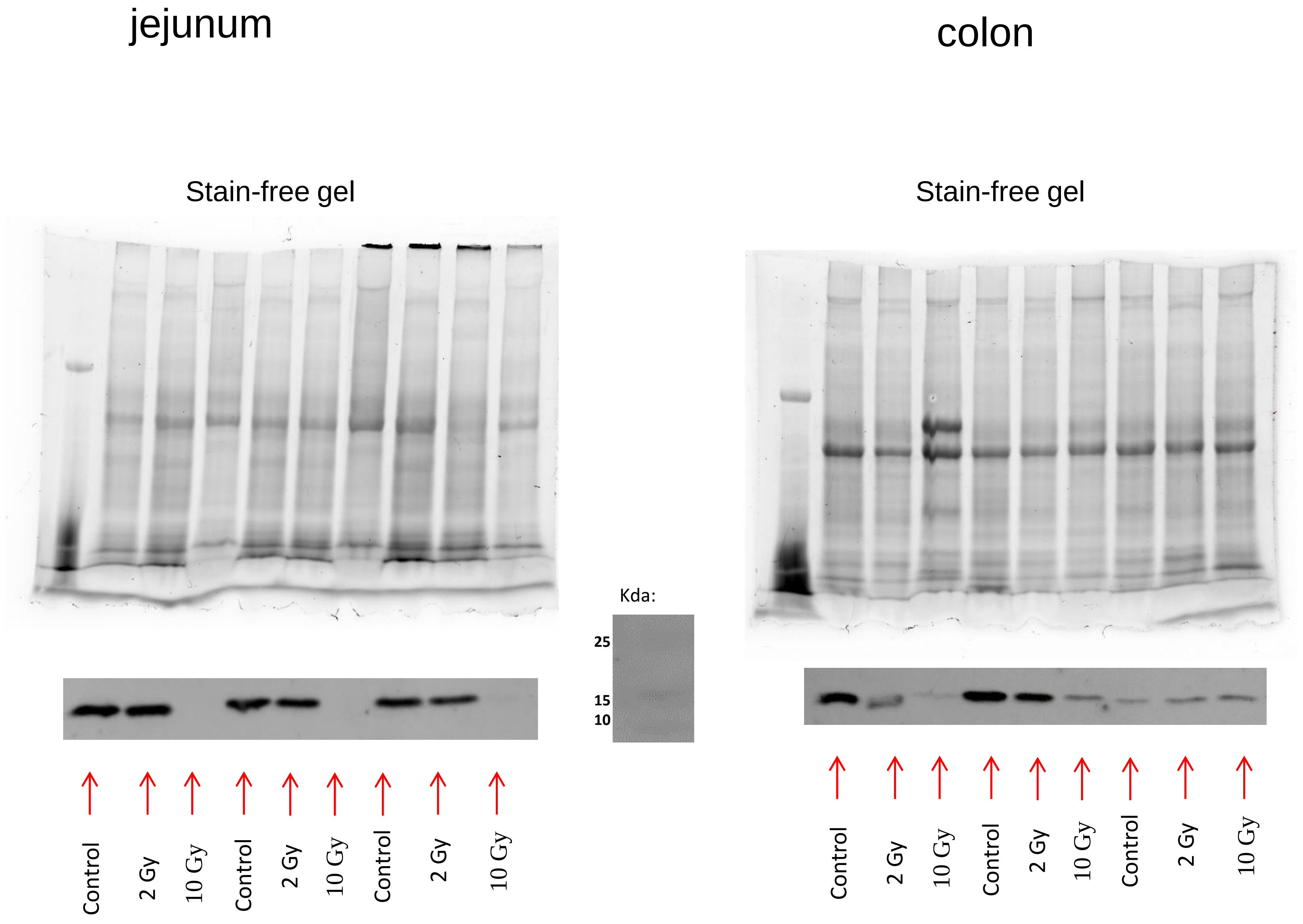

Supplement: Supplementary file 1 [file ijms-24-01753-s001.zip › ijms-2091903-supplementary.pdf]
